# Development of an ultrahigh resolution real time alpha particle imaging system for observing the trajectories of alpha particles in a scintillator

**DOI:** 10.1038/s41598-023-31748-9

**Published:** 2023-04-26

**Authors:** Seiichi Yamamoto, Masao Yoshino, Kei Kamada, Ryuga Yajima, Akira Yoshikawa, Kohei Nakanishi, Jun Kataoka

**Affiliations:** 1 Faculty of Science and Engineering, Waseda University, Tokyo, Japan; 2 New Industry Creation Hatchery Center, Tohoku University, Sendai, Japan; 3 C&A Corporation, Sendai, Japan; 4 Department of Materials Science, Graduate School of Engineering, Tohoku University, Sendai, Japan; 5 Institute for Materials Research, Tohoku University, Sendai, Japan; 6 Nagoya University Graduate School of Medicine, Nagoya, Japan

**Keywords:** Nuclear physics, Optical physics, Particle physics, Techniques and instrumentation

## Abstract

High-resolution imaging of alpha particles is required in the detection of alpha radionuclides in cells or small organs for the development of radio-compounds for targeted alpha-particle therapy or other purposes. We developed an ultrahigh resolution, real time alpha-particle imaging system for observing the trajectories of alpha particles in a scintillator. The developed system is based on a magnifying unit and a cooled electron multiplying charge-coupled device (EM-CCD) camera, combined with a 100-µm-thick Ce-doped Gd_3_Al_2_Ga_3_O_12_ (GAGG) scintillator plate. Alpha particles from an Am-241 source were irradiated to the GAGG scintillator and imaged with the system. Using our system, we measured the trajectories of the alpha particles having different shapes in real time. In some of these measured trajectories, the line shapes of the alpha particles that flew in the GAGG scintillator were clearly observed. The lateral profiles of the alpha-particle trajectories were imaged with widths of ~ 2 µm. We conclude that the developed imaging system is promising for research on targeted alpha-particle therapy or other alpha particle detections that require high spatial resolution.

## Introduction

High-resolution alpha-particle imaging is required for the distribution measurements in cells or dissected animals for the development of new radio-compounds and dosimetry for targeted alpha-particle therapy^1–3^. High-resolution alpha-particle imaging is also required for distribution measurements in mineralogical studies^4^. Moreover, alpha-particle imaging is applied to plutonium (Pu) particle detection at nuclear facilities^5,6^. To achieve real time (i.e., short time interval), high-spatial-resolution imaging for alpha particles, photodetectors have sometimes been combined with scintillators to form alpha-particle imaging systems^1,7,8^. In addition to the scintillation imaging detector, a gaseous imaging detector was also developed for a high-resolution autoradiography camera^9^. However, the spatial resolutions of these systems are insufficient to image the trajectories of alpha particles in real time. Although plastic plates such as nuclear emulsion plates or CR-39 film have achieved excellent spatial resolution and trajectories of alpha particles could be imaged, the images cannot be measured in real time. Moreover, these film-based imaging systems need post processing, such as developing the film and observing the trajectories with a microscope, which requires considerable time and labor^2^.

To improve the spatial resolution of real time radiation imaging systems, we previously combined a fiber-structure scintillator plate with a tapered fiber plate and a high-sensitivity CCD camera^10,11^. Using the previously developed system, we could achieve a spatial resolution of ~ 11 µm for alpha particles, where the trajectories of the alpha particles started to become observable^11^. However, greater improvement of the spatial resolution was difficult with a system using a tapered fiber plate.

Recently, we developed a high-resolution and high-efficiency performance evaluation system of scintillators for X-rays^12^. The imaging system could achieve a spatial resolution of up to approximately 1 µm when we combined it with micro-focus X-rays or synchrotron radiations. We noticed that the system could be applied to imaging the trajectories of alpha particles if we combined it with an appropriate scintillator plate and a high-sensitivity optical camera. Consequently, we attempted to develop an ultrahigh resolution real time alpha-particle imaging system that could clearly image the trajectories of alpha particles at the micrometer level.

## Methods

### Scintillator plate used for alpha-particle imaging

The scintillation selection is important for an imaging detector. Typically, a low-Z scintillator is suitable for an alpha particle imaging detector to obtain longer alpha-particle trajectories. However we could not find a suitable scintillator of low-Z materials with transparent and high light output that is able to image the trajectories of alpha particles. The scintillator plate selected for the imaging of alpha particles was Gd_3_Al_2_Ga_3_O_12_ (GAGG). We selected this because of its transparency, high light output, and light emission spectra suitable to a CCD camera.

The major properties of the GAGG used in our alpha-particle imaging experiments are summarized in Table 1^13^. One reason for selecting GAGG for the imaging experiments was its high light emission. To image the trajectories of alpha particles with high contrast, a higher light emission was essential. Another reason for selecting GAGG was that the maximum light emission wavelength was suitable for the maximum sensitivity of the cooled electron multiplying charge-coupled device (EM-CCD) camera used for the imaging (500–600 nm), which increased the intensity of the images of alpha particles.

**Table 1:** Main properties of scintillator plates used for imaging experiments.

Scintillator	GAGG
Density (g/cm^3^)	6.63
Maximum emission light wavelength (nm)	520
Light emission (photons/MeV)	45,000–50,000
Decay time	~ 90 ns

A Tb:GAP-Al_2_O_3_ plate^14,15^ has also been proposed as a candidate for the imaging of the trajectories of alpha particles in combination with an EM-CCD camera. However, since GAGG currently provides greater uniformity as a scintillator plate than the Tb:GAP-Al_2_O_3_ plate, we selected GAGG for the experiments.

## Developed alpha-particle imaging system

**Figure 1:**
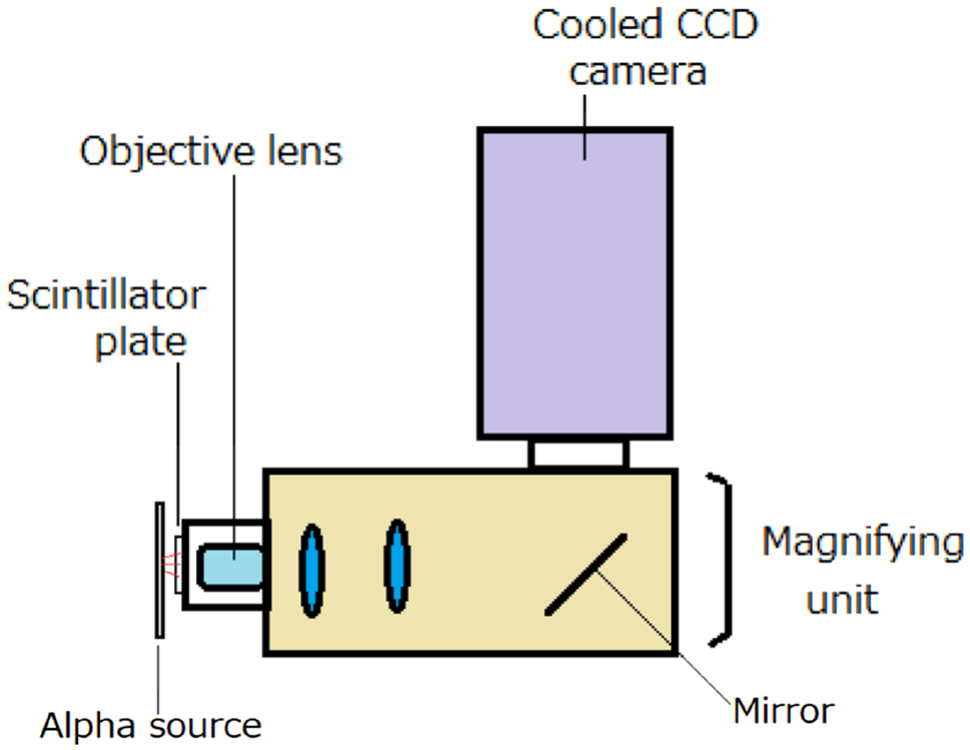
Schematic drawing of developed high-resolution alpha-particle imaging system.

A schematic drawing of the developed high-resolution alpha-particle imaging system is shown in Fig. 1. The imaging system is based on a magnifying unit and an EM-CCD camera, and it is combined with a thin GAGG scintillator plate. Alpha particles were irradiated to the GAGG scintillator plate set in front of the lens of the magnifying unit. The scintillation in the GAGG plate was magnified by the unit, reflected by a mirror in the unit, and imaged by the EM-CCD camera set above the magnifying unit.

**Figure 2:**
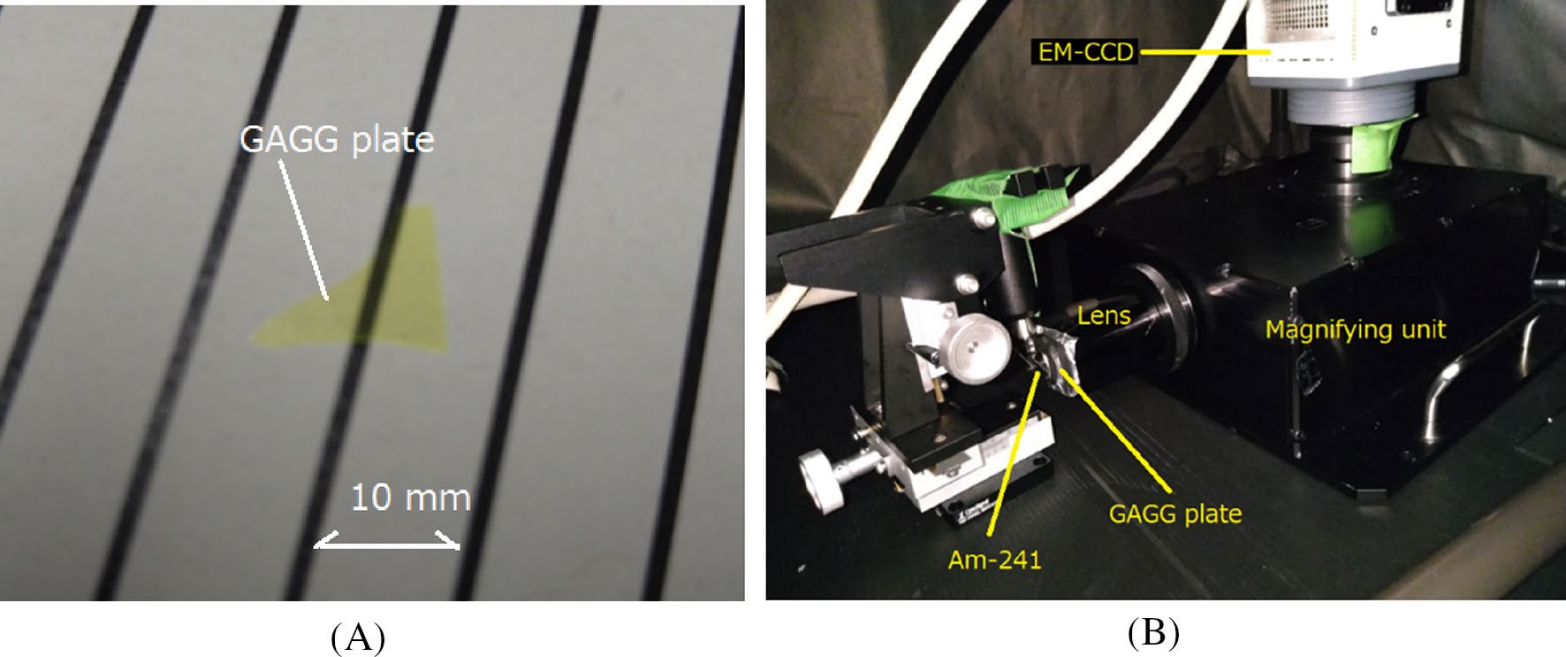
Photos of GAGG plate (**A**) and developed alpha-particle imaging system (**B**).

The GAGG plate used in the high-resolution alpha-particle imaging system was made by Tohoku University, and its original size was 10 mm × 10 mm × 0.1 mm thick. The surface of this GAGG plate was polished. A part of the scintillator (Fig. 2A) was used for the imaging because the plate’s size was much larger than the field of view (200 µm) of the imaging system.

The magnifying unit and EM-CCD camera are shown in Fig. 2B. The magnifying unit used for the imaging was a commercial one (AA51, Hamamatsu Photonics, Japan) with an attached 40 × lens objective (CFI Plan Apo Lambda 40 × , Nikon Corporation, Tokyo, Japan).

The GAGG plate was set in front of the lens objective. The camera used for the alpha-particle imaging system was a cooled EM-CCD type operating at – 65 ºC (Hamamatsu Photonics, ImagEM C9100, Japan). The pixel matrix of the EM-CCD sensor was 512 × 512. The imaging system was set inside a light-shielded black box for easy operation during the experiments.

A desktop computer was used to control the EM-CCD camera and display the measured alpha particle images from outside the black box in real time. The magnifying unit could be focused from outside the black box that made possible the precise focusing of the alpha-particle images in the GAGG plate. The field of view (FOV) of the EM-CCD was 200 µm × 200 µm with a pixel size of 0.4 µm × 0.4 µm.

## Imaging experiments

### Imaging experiments with low-energy alpha particles

We conducted imaging of the trajectories of alpha particles using the developed system. We used two alpha sources for the imaging: one was a low-energy (LE) alpha source and the other was a high-energy (HE) alpha source. The LE alpha source used for the imaging was Am-241 with a surface coating of palladium (Pd) (Type 162, JRIA, Japan), which emitted a maximum energy of ~ 2.2 MeV. Since the activity of the low-energy alpha source was high (3 MBq), it was used for imaging multiple alpha-particle trajectories in a short acquisition time. It was also used for focusing of the system because the widths of the trajectories were easier to observe in a short time. The HE alpha source used for the imaging was Am-241 without surface coating, which emitted a maximum energy of 5.5 MeV (2 kBq). Before the imaging, the energy spectra of the alpha particles were measured with these two alpha sources to quantify the energies.

### Energy spectra measurement of alpha source

Energy spectra measurement of alpha sources were measured by one of these two alpha sources set on a 1-mm-thick, 20 mm × 20 mm plastic scintillator plate (EJ-200, Eljen Technology, USA) combined with a 3-inch-diameter high-quantum-efficiency photomultiplier tube (PMT) (Hamamatsu photonics, R6233-100HA). Signals from the PMT were amplified by a standard nuclear instruments module and fed to a multi-channel analyzer (MCA) (Clear Pulse, 1125, Japan). For the measurement using an LE alpha source, the source surface was covered by a ~ 0.2-mm-thick polyvinyl tape with a small hole to decrease the number of alpha particles and thus avoid a pile-up of the system.

### Imaging experiments with low-energy (LE) alpha particles

After the LE (2.2 MeV), high-activity alpha source was set in front of the GAGG plate as shown in Fig. 1, the focus of the magnifying unit was adjusted during irradiation of alpha particles to minimize the widths of the alpha-particle trajectories observed in the images acquired by the EM-CCD camera. Since the focusing was motor-controlled by the magnifying unit, it was efficient, accurate, and reproducible.

Imaging of the EM-CCD camera was conducted with the minimum acquisition time of 500 ms (2 Hz) for ~ 10 s and the maximum image size (512 × 512 pixels). The alpha particles were irradiated to the GAGG plate from the left side ~ 5 mm from the GAGG plate’s surface as shown in Fig. 2B. No reflector was used for the GAGG plate.

### Imaging experiments with high-energy (HE) alpha particles

After the HE (5.5 MeV), low-activity alpha source was set in front of the GAGG plate, imaging of the EM-CCD camera was conducted with an acquisition time of 2 s (0.5 Hz) and the maximum image size (512 × 512 pixels). The alpha particles were irradiated to the GAGG plate from the left side ~ 5 mm from the GAGG plate’s surface. Since the activity of the alpha source was low and the FOV of the imaging system was small (200 µm × 200 µm) while the alpha source size was large (~ 15 mm in diameter), an image containing the alpha-particle trajectory was detected in ~ 10% of the acquired images. Those images containing alpha-particle trajectories were stored to the memory and evaluated.

## Image processing and evaluation methods of spatial resolution

The images recorded by the EM-CCD were processed by a software application (ImageJ)^16^. The profiles were set to the trajectories of alpha particles to evaluate the ranges and widths of the alpha particles in the images. The widths were evaluated by a Gaussian-fit function using Origin 2018b software^17^. Since the widths of the trajectories were narrow, they were used for the evaluation of the spatial resolution in full width at half maximum (FWHM) of the alpha-particle images acquired by the system.

## Results

### Energy spectra measurement of alpha source

**Figure 3:**
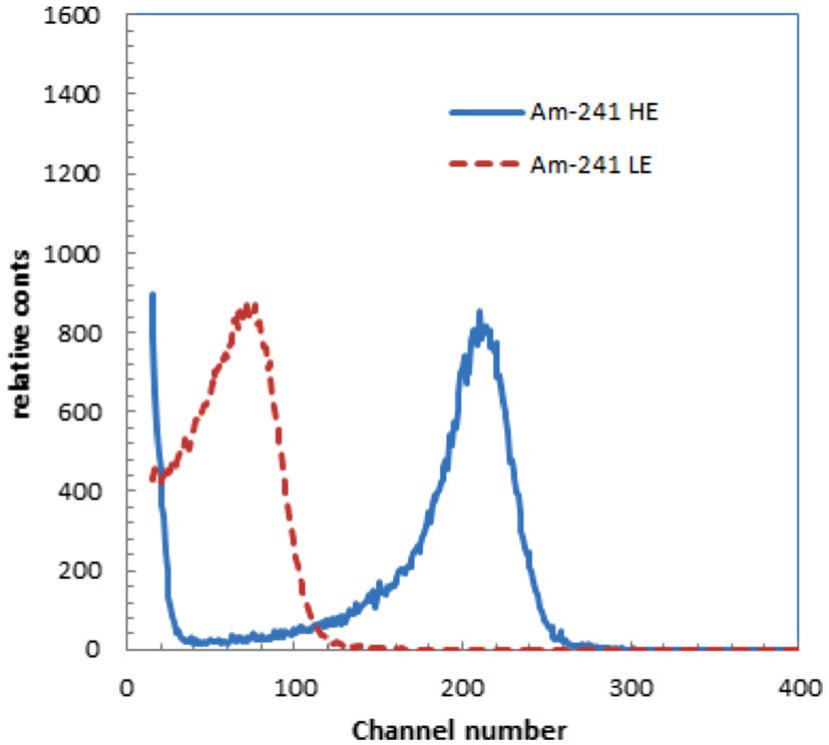
Energy spectra of two types of alpha sources used for experiments.

The energy spectra for the two types of alpha sources are shown in Fig. 3. The spectrum without surface coating has a higher energy peak (Am-241 HE) than that of the spectrum with surface coating (Am-241 LE). Assuming that the energy of the alpha source without coating (Am-241 HE) is 5.5 MeV, that with surface coating (Am-241 LE) would be ~ 2.2 MeV.

## Imaging experiments with LE alpha particles

**Figure 4:**
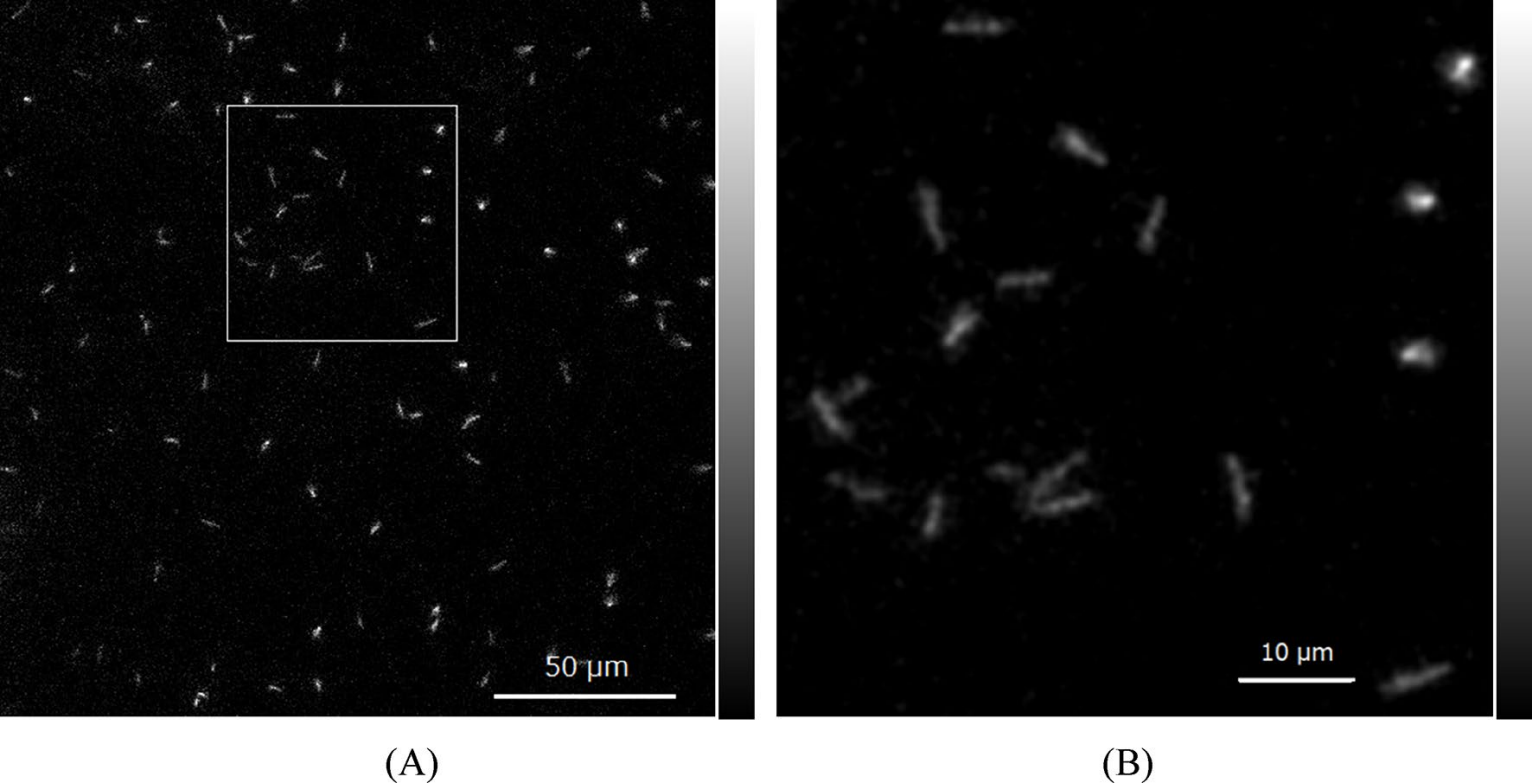
Image of alpha particles in GAGG plate measured by the developed system for LE alpha source (**A**) and magnified image (**B**) of area in square.

An image of the LE alpha particles (~ 2.2 MeV) in the GAGG plate measured by the developed system is shown in Fig. 4A. In the image, we can observe trajectories of alpha particles having different shapes, some of them round and others in line shapes.

An image of the square part in Fig. 4A magnified 3.2 times, which includes many trajectories of line-shaped alpha particles, is shown in Fig. 4B. In the image, we could observe 12–14 line-shaped trajectories. The line-shaped trajectories were the alpha particles incident in the GAGG at an angle, and the round-shaped spots with high intensity were nearly perpendicularly incident in the GAGG.

**Figure 5:**
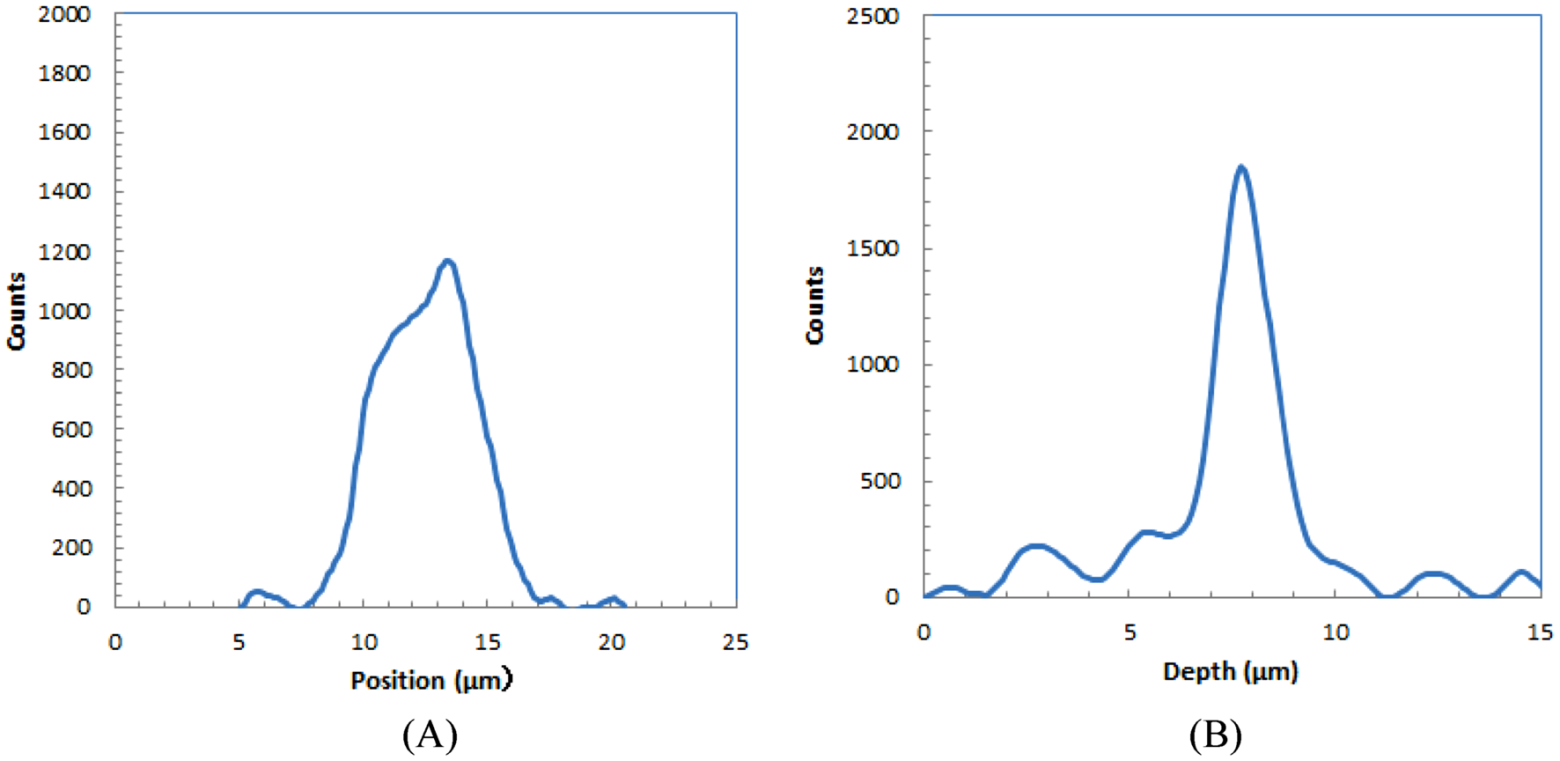
Depth (**A**) and lateral (**B**) profiles of alpha-particle trajectory for low-energy alpha source.

We plotted several profiles for the trajectories in Fig. 4B. These profiles were measured for the images with Gaussian smoothing of 0.7 µm FWHM to reduce the noise in the images. A typical profile in the longitudinal direction from alpha-particle trajectories for an LE alpha source is shown in Fig. 5A. The shape is similar to the dose distribution along the trajectory for particle ions in which a peak and a shoulder were observed because the dose distribution of alpha particles has a Bragg peak. The ranges were evaluated for the width at half of the peak for several alpha-particle trajectories.

The profile of an alpha-particle trajectory in the shorter direction is shown in Fig. 5B. The shape is similar to the typical lateral profile for a particle ion with a Gaussian shape. The widths were evaluated at half maximum for several alpha-particle trajectories.

Table 2 summarizes the average range and width evaluated for five alpha-particle trajectories imaged with the developed alpha-particle imaging system for LE alpha sources. The width was the spread of the alpha-particle images, and thus we defined this as the spatial resolution of the developed imaging system.

**Table 2:** Measured average ranges evaluated for several alpha-particle trajectories for low-energy alpha source.

Range	Width
4.8 ± 1.0 µm	1.7 ± 0.2 µm

## Imaging experiments with high-energy alpha particles

**Figure 6:**
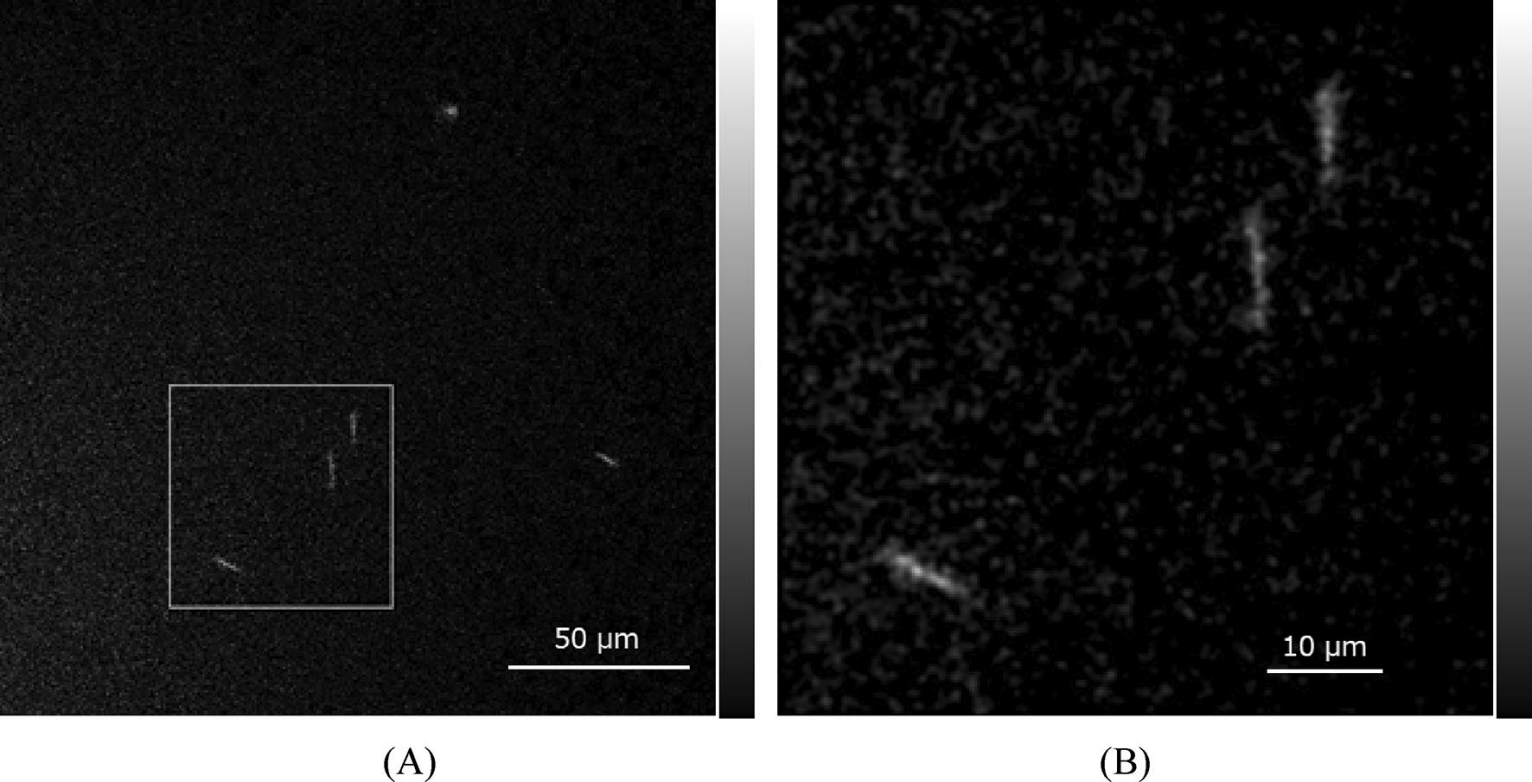
Image of alpha particles in the GAGG plate measured by the developed system for HE alpha source (**A**) and magnified image (**B**) of the area in the square.

An image of the HE alpha particles (~ 5.5 MeV) in the GAGG plate measured by the developed system combining four frames is shown in Fig. 6A. In the image, we can observe several trajectories from alpha particles with different shapes. Among these alpha-particle trajectories, some long line shapes were included.

Figure 6B shows an image of the square part in Fig. 6A magnified 3.2 times. In the image, we could observe three line-shaped trajectories from alpha particles.

**Figure 7:**
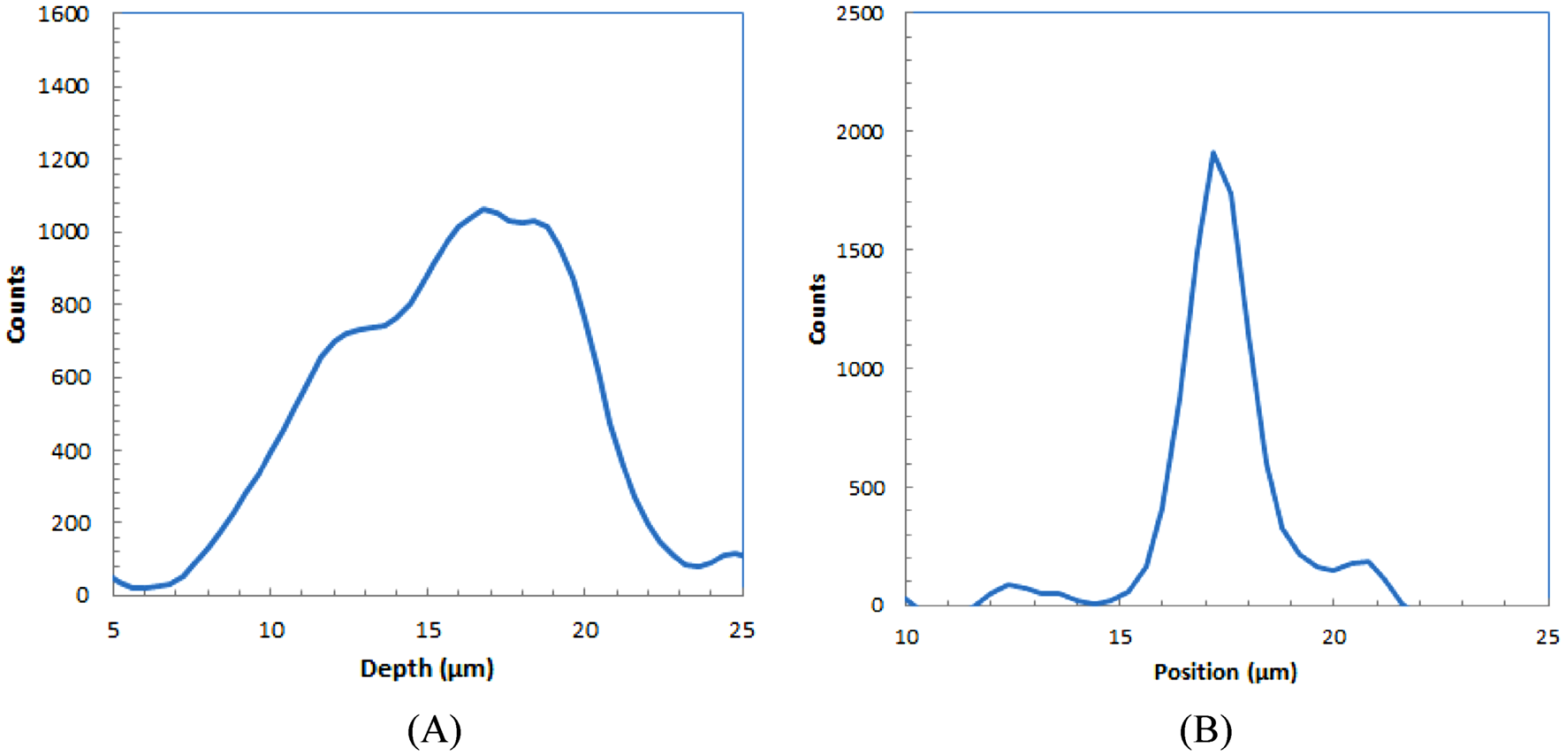
Depth (**A**) and lateral (**B**) profiles of alpha-particle trajectory for HE alpha source.

The profile of an alpha-particle spot in the longitudinal direction for a high-energy alpha source is shown in Fig. 7A. The shape is similar to the typical depth profile for particle ions in which a peak and a shoulder are observed. The ranges were evaluated for the width at half of the peak for several alpha-particle trajectories.

The profile of an alpha-particle spot in the shorter direction is shown in Fig. 7B. The shape is similar to the typical lateral profile for a particle ion with a Gaussian shape. The widths were evaluated at half maximum for several alpha-particle trajectories.

Table 3 summarizes the range and width evaluated for five alpha-particle trajectories imaged with the developed alpha-particle imaging system for an HE alpha source.

**Table 3:** Measured average ranges evaluated for several alpha-particle trajectories for HE alpha source.

Range	Width
9.5 ± 1.5 µm	2.1 ± 0.3 µm

The range was for the angled incident of alpha particles, so the actual length was longer. The simulated range by Geant4^18^ for 5.5-MeV alpha particles from Am-241 in GAGG was 13.8 µm. Assuming the average incident angle is 45º, the average measured range would be calculated as 9.8 µm, a similar value to the measured range listed in Table 3.

## Discussion

We could develop an alpha-particle imaging system with much higher spatial resolution than those of systems developed previously^10,11^. With its higher spatial resolution, the system can visualize the alpha-particle trajectories clearly in real time. Compared with our previously developed tapered fiber imaging system combined with a GAP-Al_2_O_3_ plate^11^, the spatial resolution was ~ 5 times higher and the trajectories of the alpha particles could be imaged in a line shape. One reason that such high resolution could be achieved was the use of a magnifying unit. The unit could magnify the image set in front of the objective lens if the focus were precisely adjusted. Since most of the alpha-particle trajectories were located at a shallow depth (~ 10 µm) in the GAGG plate, they could be observed at high resolution because precise focusing was possible within this depth. Focusing is inherently a difficult task for a magnifying unit, but it was relatively easy with our system because it features motor control of the distance from the subject to the lens.

Another reason for achieving high resolution was the use of a GAGG plate. The light emission of GAGG was large as listed in Table 1, and the maximum emission light wavelength was well fitted to the sensitivity of the EM-CCD camera used for the imaging. With this combination of the emission wavelength of GAGG and the sensitivity of the EM-CCD camera, the scintillation light produced by alpha particles in the GAGG plate could be imaged at high intensity. The high intensity of the spots increased the contrast of the alpha-particle images, and the trajectories of alpha particles could be observed clearly.

The advantage of the developed alpha-particle imaging system is its much higher spatial resolution than those achieved by previously developed alpha-particle imaging systems^10,11^. The spatial resolution of the developed alpha-particle imaging system (~ 2 µm) was better than that of a film-based imaging system using CR-39^2^, and it closely approached the high-resolution images of alpha particles obtained with a nuclear emulsion plate. With the developed system, we will be able to image the trajectories of alpha particles emitted from a single cell containing alpha-emitting radionuclides or a small particle of an alpha-emitting radionuclide such as PuO_2_. Using our system, it may be possible to capture images of alpha-particle trajectories showing radial spreading from the emitting cells or particles.

Another advantage of the developed alpha-particle imaging system is its real time (i.e., short time interval) imaging capability. With our developed system, high-resolution images of alpha-particle trajectories can be observed with 500-ms intervals. Therefore, the temporal changes in subjects such as cells can be measured. Furthermore, the time activity curves can be measured from the time sequential images of alpha particles, which may provide information on the physical or physiological decay of the subjects.

The background counts of the developed alpha particle imaging system are important for low-level imaging applications such as mineralogical studies. The background counts from the alpha particles emitted from Gd-152 were negligible because the half-life of Gd-152 is very long (10^14^ years) and the volume of GAGG in the field of view (FOV) was very small (10^–6^ cc). Background from gamma photons, X-rays, or beta particles was not detected by the developed imaging system due to the low intensity per pixel for these radiations.

## Conclusions

We have successfully developed a high-resolution real time alpha-particle imaging system that can clearly observe the trajectories of alpha particles. The spatial resolution calculated from the width of the lateral profiles of the alpha particle trajectories in the images was ~ 2 µm. Accordingly, the developed imaging system is promising for research on targeted alpha-particle therapy or other alpha emitter detections that require high spatial resolution.

## Data Availability

All data generated or analyzed during this study are included in this published article.
